# *pyHeart4Fish*: Chamber-specific heart phenotype quantification of zebrafish in high-content screens

**DOI:** 10.3389/fcell.2023.1143852

**Published:** 2023-04-11

**Authors:** Viviana L. Vedder, Tobias Reinberger, Syed M. I. Haider, Luis Eichelmann, Nadine Odenthal, Salim Abdelilah-Seyfried, Zouhair Aherrahrou, Maximilian Breuer, Jeanette Erdmann

**Affiliations:** ^1^ Institute for Cardiogenetics, University of Lübeck, Lübeck, Germany; ^2^ DZHK (German Centre for Cardiovascular Research), Partner Site Hamburg/Kiel/Lübeck, Lübeck, Germany; ^3^ University Heart Centre Lübeck, Lübeck, Germany; ^4^ Faculty of Mathematics and Natural Sciences, Institute for Biochemistry and Biology, University Potsdam, Potsdam, Germany

**Keywords:** Zebrafish, drug screen, *pyHeart4Fish*, heart, drug effects, development, python GUI application, cardiovascular disease

## Abstract

Cardiovascular diseases (CVDs) are the leading cause of death. Of CVDs, congenital heart diseases are the most common congenital defects, with a prevalence of 1 in 100 live births. Despite the widespread knowledge that prenatal and postnatal drug exposure can lead to congenital abnormalities, the developmental toxicity of many FDA-approved drugs is rarely investigated. Therefore, to improve our understanding of drug side effects, we performed a high-content drug screen of 1,280 compounds using zebrafish as a model for cardiovascular analyses. Zebrafish are a well-established model for CVDs and developmental toxicity. However, flexible open-access tools to quantify cardiac phenotypes are lacking. Here, we provide *pyHeart4Fish*, a novel Python-based, platform-independent tool with a graphical user interface for automated quantification of cardiac chamber-specific parameters, such as heart rate (HR), contractility, arrhythmia score, and conduction score. In our study, about 10.5% of the tested drugs significantly affected HR at a concentration of 20 µM in zebrafish embryos at 2 days post-fertilization. Further, we provide insights into the effects of 13 compounds on the developing embryo, including the teratogenic effects of the steroid pregnenolone. In addition, analysis with *pyHeart4Fish* revealed multiple contractility defects induced by seven compounds. We also found implications for arrhythmias, such as atrioventricular block caused by chloropyramine HCl, as well as (R)-duloxetine HCl-induced atrial flutter. Taken together, our study presents a novel open-access tool for heart analysis and new data on potentially cardiotoxic compounds.

## 1 Introduction

For almost 2 decades, cardiovascular diseases (CVDs) have remained the leading cause of death worldwide (WHO, 2017; WHO, 2019). Among CVDs, congenital heart diseases (CHDs) are the most common human congenital defect (van der Linde et al., 2011; Virani et al., 2021). Exposure to several chemicals and pharmaceuticals during pregnancy and early infancy negatively impacts development. Studies estimate a prevalence of 1%–4% of congenital defects caused by adverse drug effects (Hernandez et al., 2012; Colvin and Walters, 2016; Uguz, 2020). Approximately 55%–90% of women receive at least one prescription drug during pregnancy (Huybrechts et al., 2018; Dathe and Schaefer, 2019). However, data on the specific prenatal and postnatal teratogenicity of drugs and their effect on cardiovascular development is scarce and often retrospective (Halpern et al., 2019).

*In-vivo* drug testing for developmental toxicity is predominantly performed in mammalian models, such as mouse, rat, and rabbit models (Barrow, 2018). Due to low scalability, these tests are expensive, labor-intensive and require a large number of animals, thereby raising ethical concerns. *In-vitro* drug screens have high scalability, do not require the use of animals, and raise few ethical concerns; however, they lack complex physiological context. Therefore, *in-vivo* high-throughput screens using small model organisms, such as *Caenorhabditis elegans*, *Drosophila melanogaster* and *Danio rerio* (zebrafish), have been developed. They are more cost-effective and have greater scalability than mammalian models while retaining the body’s complex physiology (Piersma, 2004; Patton et al., 2021). In 2000, Peterson *et al.* published the first high-content screen in zebrafish (Peterson et al., 2000). Following this publication, zebrafish have become a widely used vertebrate model for drug screens and human diseases. Several successful *in-vivo* screens in zebrafish have been performed, and the findings have transitioned into clinical trials (Cully, 2019; Zhao et al., 2019). The small size, optical transparency, rapid *ex-utero* development, and robustness of zebrafish make them an ideal model for *in-vivo* high-content screening (Fang and Miller, 2012; Wilkinson and van Eeden, 2014). Additionally, easy genetic manipulation has yielded several transgenic zebrafish lines, such as Tg(*fli1a:nEGFP*)^y7^ and Tg(*myl7:eGFP*)^twu34/+^, that permit the *in-vivo* observation of the highly conserved cardiovascular development through the use of fluorescence labels (Asnani and Peterson, 2014; Staudt et al., 2014; Bowley et al., 2021). In compliance with the current EU legislation, zebrafish embryos under 5 days post-fertilization (dpf) comply with the 3R principle of reduce, refine, and replace (Russell and Burch, 1959; Tannenbaum and Bennett, 2015). Further, 71.4% of human genes have at least one orthologue in zebrafish and more than 80% of human drug targets have zebrafish orthologues (Gunnarsson et al., 2008; Vilella et al., 2009; Howe et al., 2013).

The heart is one of the first organs to form during vertebrate organogenesis (Buckingham et al., 2005; Bulatovic et al., 2016). Cardiogenesis is a highly conserved process that starts at 3 weeks of gestation in humans and 5 h post-fertilization (hpf) in zebrafish (Olson, 2006; Brown et al., 2016). Briefly, two bilateral heart fields, derived from the precardiac mesoderm, develop during gastrulation and fuse to form the primary heart tube (Wessels and Sedmera, 2003). Progressing development divides the tube into two chambers, the atrium and ventricle, creating a polarity of the vertebrate heart with the atrium at the inflow tract (Olson, 2006). Dominant pacemaker activity is observed at the inflow tract, facilitating unidirectional blood flow (Hirota et al., 1979; Moorman et al., 1998). The single atrium and ventricle present the basic blueprint for the teleost heart. Additional looping and partitioning of the chambers form the mammalian four-chambered heart. Teleosts, such as zebrafish, do not undergo the additional chamber septation and instead have a bulbus arteriosus that regulates blood pressure. However, signaling pathways and specialized cell types and structures, such as pacemaker cells, valves and coronary vasculature, are conserved (Staudt et al., 2014; Bowley et al., 2021). All these factors make zebrafish an excellent model for studying cardiovascular developmental toxicity. In humans, the heart is not completely developed at birth (Buijtendijk et al., 2020). Thus, its development can be affected by genetic and environmental factors throughout gestation and postnatal development.

In 2020, Gierten et al. published the *HeartBeat* software, an open-access tool that can be utilized to analyze data generated with the ACQUIFER Imaging Machine in a high-throughput manner (Gierten et al., 2020). This tool offers a reliable readout of heartbeat frequencies for one or multiple fish per well. However, no tool simultaneously analyzes several chamber-specific heartbeat phenotypes in fish embryos, such as size, contractility, arrhythmia, and conduction defects. Therefore, novel tools were needed to further advance the high-throughput analysis of heartbeat phenotypes.

Here, we report the results from a high-content screen of 1,280 compounds for cardioactive effects in zebrafish. Two transgenic lines were utilized to exclude compounds with line-specific effects. Hits with repeatable effects were analyzed using our newly-developed tool, *pyHeart4Fish*. Currently, *pyHeart4Fish* can quantify heart rate (HR), heart size (HS), ejection fraction (EF), relative contractility, maximal (max.) and minimal (min.) chamber size (dilation and contraction), and provide an arrhythmia and conduction defect score. Quantifying our hits revealed that seven compounds produced chamber-specific contraction and dilation defects, two compounds reduced overall HS, and two additional compounds induced arrhythmias. Altogether, this study provides a robust drug screen with a reliable novel tool for in-depth functional phenotyping of the heart.

## 2 Material and methods

### 2.1 Ethics statements

Zebrafish husbandry and experiments were performed following German animal welfare legislation. Under German and European legislation, this work does not involve research with animals. Zebrafish embryos were obtained from closed stocks at the Fraunhofer Institute for Marine Biology (EMB), Lübeck, Germany, under the supervision of the local representative of the animal welfare agency.

### 2.2 Zebrafish husbandry and crossing

Adult zebrafish were kept according to standard protocols with 12/12 h light/dark cycle at 28°C (Westerfield, 2000). Transgenic Tg (*fli1a:nEGFP*)^y7^ zebrafish were obtained from the European Zebrafish Resource Center (EZRC), and Tg(*myl7:eGFP*)^twu34/+^ were kindly provided by the University Potsdam, Institute for Biochemistry and Biology.

Eggs were collected from pairwise mating. Water was exchanged before the dividers were removed from each tank. Then, eggs were collected after 1 h of mating to minimize variability in development. Subsequently, unfertilized eggs and debris were removed before embryos were incubated overnight at 28°C in egg water.

### 2.3 Drug exposure

Embryos were dechorionated at 21 hpf using 0.22 mg/ml pronase (Merck Chemicals) for 12 min. Then, embryos were carefully washed three times with egg water and transferred into a 96-deep-well plate. After incubation at 28°C for 3 h, excess water was removed with a glass capillary and exchanged with 100 µL/well of 20 µM compound solution from the Prestwick Chemical Library (Prestwick Chemicals, France) in 0.2% DMSO (v/v) in egg water. The small-molecule library consists of 1,280 compounds dissolved in 100% DMSO. Subsequently, embryos were exposed to compounds for 24 h at 28°C in the dark to enable testing of light-sensitive compounds. Solutions were removed at 48 hpf before wells containing embryos were washed three times with system water. Then, treated embryos were transferred into 12-well plates containing 1 ml 0.03% tricaine in system water (Sigma-Aldrich, Germany). Testing different concentrations (5–80 µM) of compounds and their respective controls followed the above protocol.

### 2.4 Agarose molds in 96-well plates

Agarose molds in flat-bottom 96-well plates were generated using a previously published 3D-printed orientation tool (Wittbrodt et al., 2014). Each well was filled with 65 µL of 1.5% agarose (Biozyme) dissolved in 0.03% buffered tricaine using a multi-channel pipette. Then, the orientation tool was inserted to create the molds and removed after the agarose was completely cool. Subsequently, embryos were transferred into 96-well plates containing the agarose orientation molds using 50 µl of tricaine (Wittbrodt et al., 2014). Zebrafish were oriented under a stereomicroscope before imaging.

### 2.5 Image acquisition

Oriented embryos in 96-well plates were automatically imaged using an ACQUIFER Imaging Machine (ACQUIFER Imaging, Germany) widefield microscope. Z-stacks of 10 slices (dZ = 15 µm) were acquired in brightfield (BF) and 470 nm channels with a 4×0.13 numerical aperture objective. The focal plane was detected in the 470 nm channel using a course and secondary fine software autofocus. BF integration time was fixed at 20 ms exposure time and 55% LED intensity. The exposure time for the 470 nm channel was set to 10 ms with a 10% LED intensity. Heartbeat videos of 6 s at 6 frames/second (f/s) were acquired with a time-lapse at a 0 s interval in the focus plane acquired after secondary autofocus using the previously described integration and exposure settings with a 10×0.25 numerical aperture objective.

### 2.6 Data analysis

#### 2.6.1 Primary HR screen

Acquired image series were preprocessed and analyzed using the previously published *HeartBeat* software v.2.1 and provided scripts (Gierten et al., 2020). For increased comparability between experiments, each quantitative HR value (beats per minute) was divided by the mean of the DMSO controls from the respective experiment. This procedure minimizes variation and creates specific fold-change values that are z-score normalized in the next step. A 90% CI was used to reduce the probability of false-negative results. Finally, data from compounds with insufficient N and known aquatic toxicity were removed.

#### 2.6.2 Analysis with *pyHeart4Fish*

To gain deeper insights into the toxic effects of compounds on heart morphology and function, Tg(*myl7:eGFP*)^twu34/+^ zebrafish embryos were treated with hit compounds according to the protocol and analyzed using *pyHeart4Fish*. The Python-based tool provides graphical user interphase (GUI; implemented in the tkinter package) and accepts several data file formats, such as image series (TIF or PNG files), videos (AVI or MP4 files) or microscope-specific formats (e.g., CZI files from ZEISS). In brief, images/frames of videos are processed using the OpenCV library (cv2), Python imaging library, and NumPy. Hearts are analyzed chamber-specifically *via* the user-assisted region of interest (ROI) selection. Cardiac morphology parameters (HS and shape) are extracted as the median of the first six images/frames. Raw data of heartbeat curves for the atrium and ventricle are derived from the sum of pixel intensities and normalized chamber areas. Frequencies (in s^-1^) of atrium and ventricle contraction are obtained by applying sine-curve fitting or fast Fourier transformation (FFT) using the SciPy modules “optimize.curve_fit” and “fft.rfft/fft.rfftfreq”, respectively (Virtanen et al., 2020). Curve fitting is performed with a maximum of 30 iterations while increasing the frequencies of the fitted sine function. The best fit is selected using the correlation coefficient between the raw data and the fitted function implemented in the NumPy package. A fitted sine function and fitting score are also determined for a truncated signal (first quarter), and the best-fitted sine function is kept to account for an unstable heartbeat signal. In the case of FFT, the first harmonic of the FFT signal is used as the frequency of the atrium or ventricle contraction. Analysis with *pyHeart4Fish* provides heartbeat videos in GIF format, heartbeat curves as PNG/PDF files and a data sheet containing meta information and cardiac parameters. The Pearson correlation coefficient r (as implemented in the Python module scipy. stats.pearsonr) was used to evaluate the reliability of *pyHeart4Fish.* Three correlation analyses of HR and other parameters were performed: 1) *pyHeart4Fish* vs. manual quantification (50 zebrafish), 2) *pyHeart4Fish* user1 vs. *pyHeart4Fish* user2 (intra-assay variability, 85 zebrafish), and 3) a pairwise comparison of experimental replications (intra-assay variability, 561 zebrafish, 2–13 replications) (Supplementary Figure S6). Extreme outliers with more than a 3-fold y standard deviation (SD) from the regression curve were not considered when calculating the Pearson correlation coefficient. Besides the chamber-specific module, *pyHeart4Fish* also provides a fully automated quantification of HRs of BF zebrafish movies (BF module). The heart area is selected automatically. The HR is determined from the values of pixel intensities per frame, averaging 10 pixels with the highest SD over all the frames. Curve fitting and FFT were performed as described above.

#### 2.6.3 Toxicity score

Acquired data from sagittal BF videos on developmental delay, pericardial edemas, cardiac arrest and z-score normalized HR were used to calculate a toxicity score (ToxScore) to assess the dose-dependency of the toxic effects. The ToxScore was calculated as follows:

$$ToxScore=\frac{n\;pericardial\;edema}{n\;total}+\frac{n\;developmental\;delay}{n\;total}+\left(1=HR\pm 1.64\right)+\frac{n\;cardiac\;arrest\times 2}{n\;total}$$

Embryos that were deceased and not intact were assigned a max. ToxScore value of 5.

## 3 Results

### 3.1 High-content screening for heartbeat phenotypes

**FIGURE 1:**
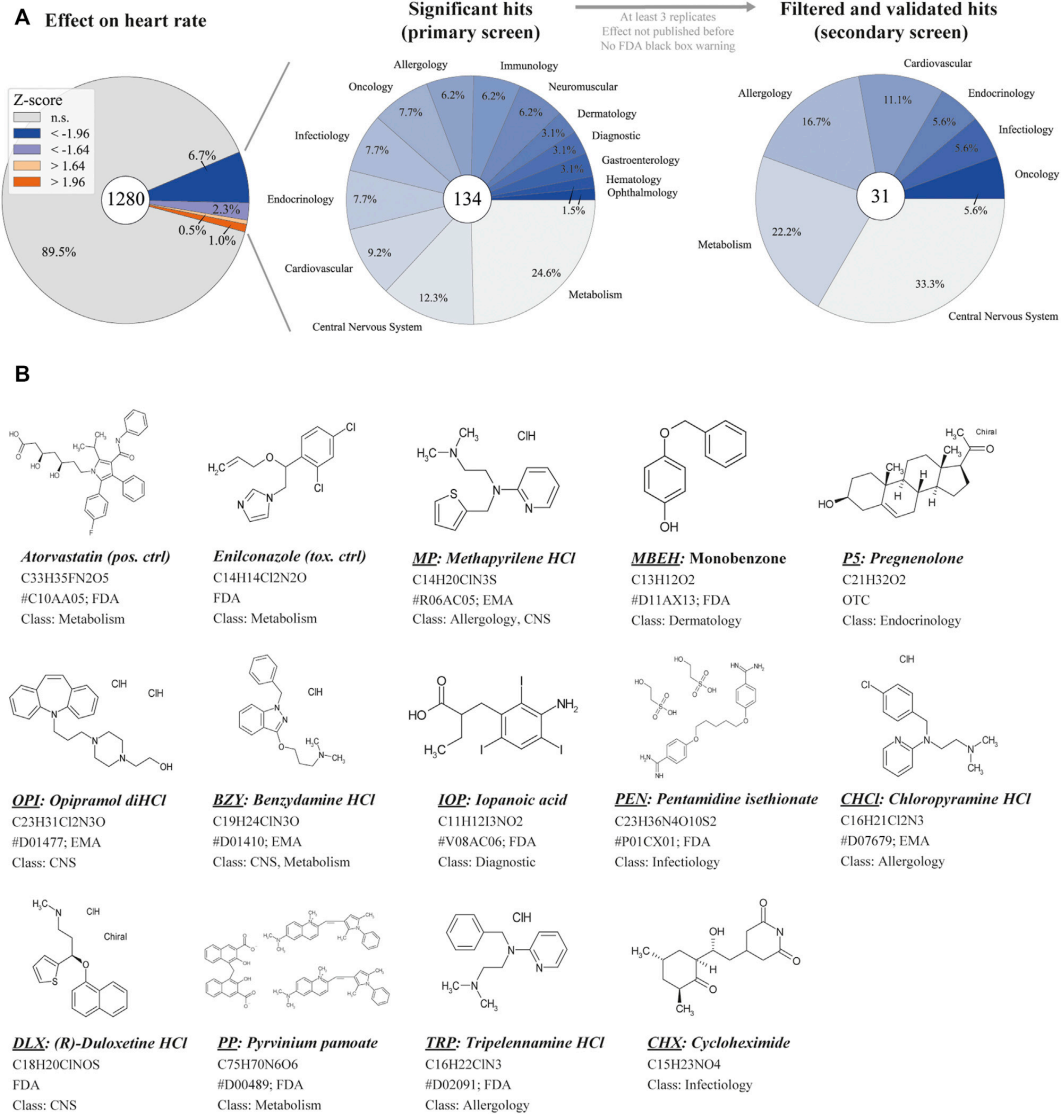
Overview of primary screening **(A)** High-content screening results for z-normalized heart rate data, obtained by analysis with *HeartBeat* software (v.2.1). Z-scores above or below ±1.96 are considered significant with a 95% CI. For the primary screen, effect sizes of a 90% CI (±1.64) were considered to reduce false-negative results. The center pie-chart shows the therapeutic classes of the 134 hits (90% CI). The therapeutic classes of the remaining 31 hits after filter application is shown on the right. **(B)** The structure of selected hit compounds with cardioactive properties. Class = therapeutic class; CNS = central nervous system; EMA = European Medicines Agency; FDA = Food and Drug Administration; HCl = Hydrochloride; OTC = over-the-counter drug; anatomical therapeutic chemical (ATC) classification are displayed (indicated by #) if available.

Based on a previously established high-throughput screening pipeline, we performed a high-content drug screen in zebrafish for cardiovascular modulators (Poureetezadi et al., 2014; Gierten et al., 2020; Westhoff et al., 2020). The Prestwick Chemical Library consisting of 1,280 compounds was screened for cardioactive compounds. About 98% of the library’s compounds are approved by the Food and Drug Administration (FDA), European Medicines Agency (EMA) or other regulatory agencies. The drug library was screened at a concentration of 20 µM for 24 h until 48 hpf using the transgenic zebrafish line Tg(*fli1a:nEGFP*)^y7^. Subsequently, BF images and videos were captured at 2 dpf to assess gross morphology. HR was analyzed with the previously published tool *HeartBeat* (v.2.1) to identify potential hit compounds. A 90% CI threshold for effect sizes of ±1.64 was used to reduce false-negative results. The primary drug screen identified 134 compounds that significantly affected HR (Figure 1A). Performing a systematic database and literature research resulted in the removal of 50 compounds with known side effects on HR. The Open Targets platform and the Mayo Clinic Drugs and Supplements Database were systematically searched for compound names and common synonyms (Drugs and Supplements, 2022; Open Targets, 2022). The Medical Subject Headings (MeSH) terms “compound name” and “heart rate” were applied to NCBI PubMed literature research before the first five pages of publications sorted by relevance were screened for relevant information. Furthermore, compounds were filtered for unknown targets (*n* = 3), FDA black box warning (*n* = 6) and aquatic toxicity (*n* = 4). Compound-induced lethality at the tested concentration resulted in a varying number of replicates n). Therefore, compounds with *n* < 3 were excluded (*n* = 7).

In the second step, treatment of Tg(*fli1a:nEGFP*)y7 with the remaining compounds was repeated, and compounds with non-repeatable effects were removed. Next, a second transgenic line, Tg(*myl7:eGFP*)^twu34/+^, was treated with potential hit compounds and the effects were recorded to gather more information on heart morphology, function and toxicity (*n* = 4–11, depending on compound lethality). This approach prevents the detection of line-specific effects. Finally, we focused on 13 of the remaining 31 compounds that displayed significant effects on diverse functional heart phenotypes at 20 µM (Figure 1B).

### 3.2 *pyHeart4Fish*

Despite ever-improving *in-vivo* imaging technologies for the characterization of cardiac phenotypes, it is crucial to detect even subtle cardiac side effects of drugs, especially if the drugs are used during pregnancy. With improved imaging technologies, large data management systems and semi-automated or automated data analysis became paramount. Therefore, we developed *pyHeart4Fish*, a Python-based tool specifically designed to analyze cardiac phenotypes in fish in a chamber-specific manner. This versatile tool can be used with various data types independent of a microscope or imaging system.

*pyHeart4Fish* can analyze HR, HS, contractility, and EF, as well as arrhythmia and conduction defects from several data types, such as image series and video files (Supplementary Figure S1). This tool provides two frequency calculations. The first heartbeat frequency is derived from a fitted sine function and is more suitable for frame rates ≥9 f/s. The second heartbeat frequency is derived from a FFT and is suitable for lower frame rates such as 6 f/s. However, capturing videos at higher frame rates is recommended, especially for precise arrhythmia detection. Nevertheless, it is advised that arrhythmia types are confirmed manually, as was done in this study. To assess *pyHeart4Fish,* we compared manual and software-based counts of chamber-specific HR for 50 randomized, blinded heartbeat videos (Pearson’s r = >0.99 for both atrium and ventricle; Figure 3A and Supplementary Table S1). Analysis of 85 blinded heartbeat videos by two independent users was correlated to assess the tools’ intra-specific variability (Pearson’s r = 1 for atrium and ventricle; middle panel in Figure 3A). To analyze inter-specific variability, 2–13 replicates of control and compound treatments were correlated (Pearson’s r atrium = 0.81, ventricle = 0.86; lower panel in Figure 3A). For the correlation analyses of the remaining parameters see Supplementary Figure S6. The high correlation between manual and automatic measurements and intra- and inter-specific variability demonstrates the robustness and high accuracy of *pyHeart4Fish.*

**FIGURE 2:**
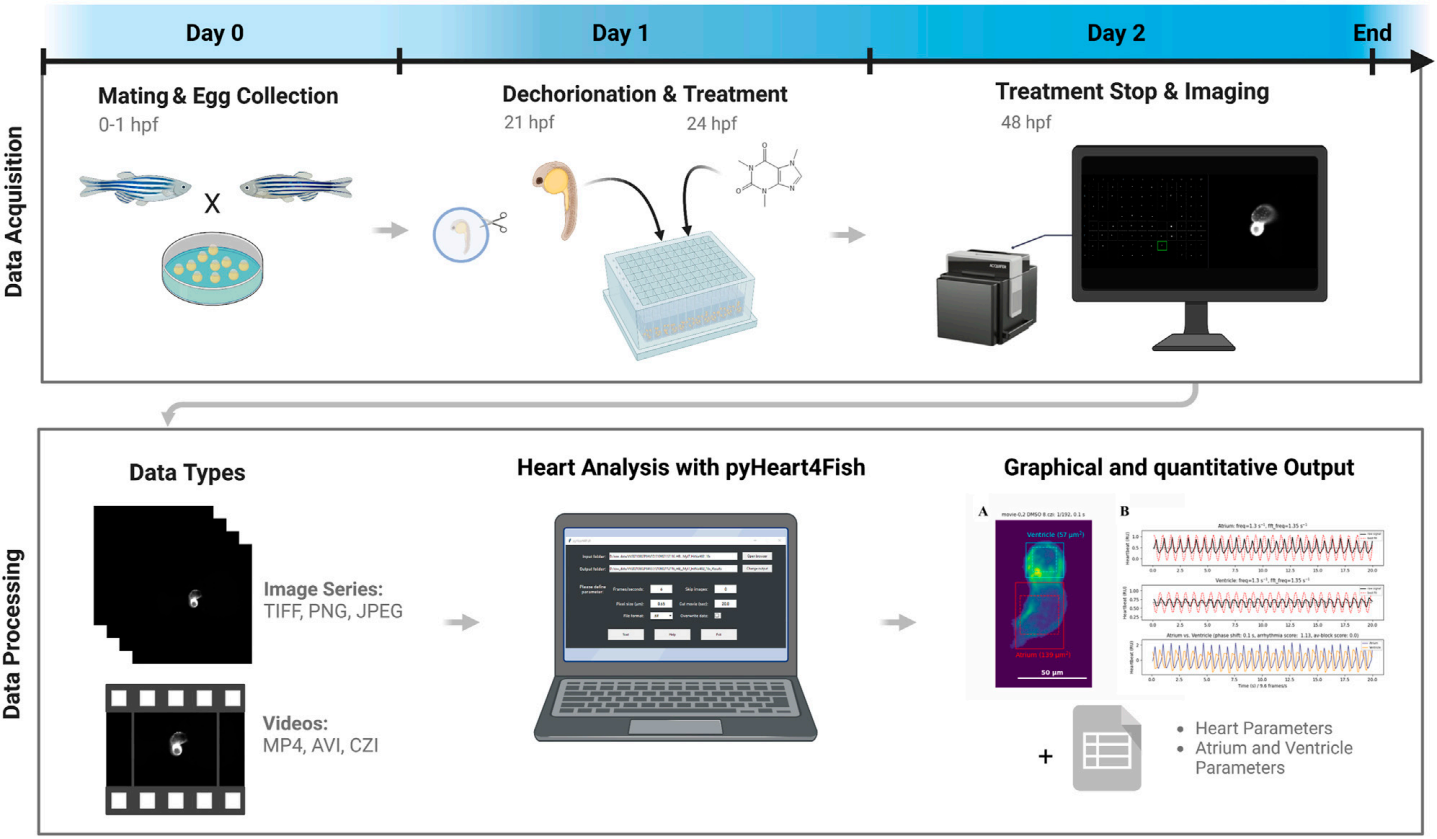
Screening and data analysis workflow. Eggs were collected after 1 h of pair- or group-wise mating before being incubated overnight. At 21 h post-fertilization (hpf), embryos were dechorionated, then at 24 hpf were treated with compounds from the Prestwick drug library. Treatment was stopped at 48 hpf, and fish were mounted in orientation plates to visualize fluorescent hearts and imaged with an ACQUIFER imaging machine. Displayed data types can be quantified using the *pyHeart4Fish* GUI and result in a graphical output in the form of GIFs and PNGs, as well as data sheets with additional meta and quantitative data. This figure was created with BioRender.com.

**FIGURE 3:**
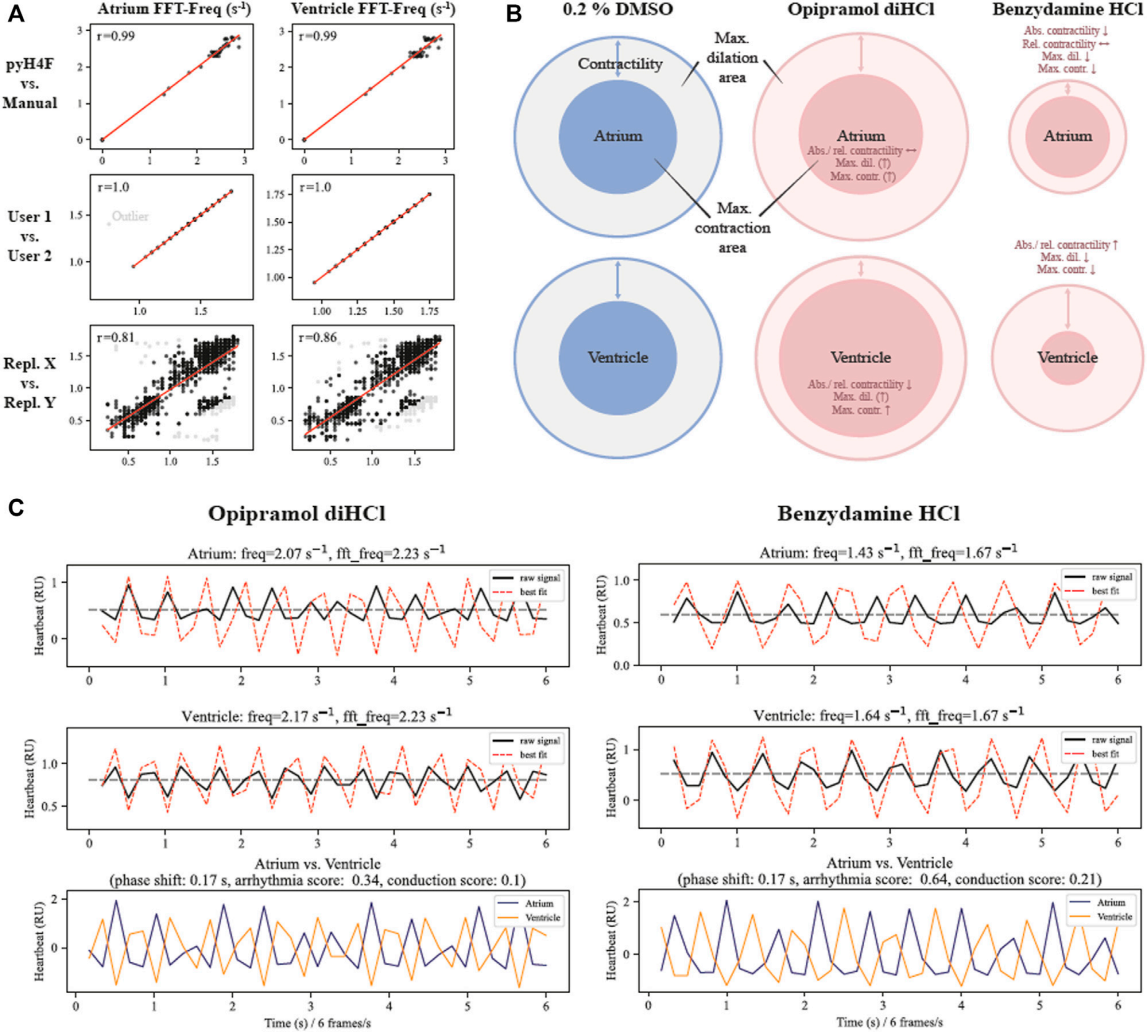
*pyHeart4Fish* validation and interpretation guide **(A)** Pearson correlation analysis for manual and semi-automated heart rate measurements in atrium and ventricle using *pyHeart4Fish* (pyH4F) vs. manual determination (50 zebrafish), user-dependent determination (85 zebrafish), and pairwise comparison of replicates (561 zebrafish, 2–13 replications). Outliers (shown in gray) with more than 3-fold y standard deviation from the regression curve were not considered when calculating the Pearson correlation coefficient. **(B)** An interpretation guide for max. dilation and max. contraction data obtained with *pyHeart4Fish*. The smallest circles indicate max. contraction and circles larger than these indicate dilation. Blue represents controls and orange represents treated hearts. The arrows indicate the difference between min. and max. chamber size in treated fish. The displayed examples demonstrate increased max. dilation with decreased max. contraction after opipramol diHCl exposure, and reduced max. dilation and contraction in the atrium of benzydamine HCl-treated embryos **(C)** A representative raw data output of *pyHeart4Fish* analysis. The overlay of raw-signal in combination with the arrhythmia score, and conduction score shows there are no conduction defects between the atrium and ventricle in opipramol diHCl- and benzydamine HCl-treated embryos. Black line = raw signal; red dotted line = fitted sine function; freq = frequency estimated using the sine function; fft_freq = fast fourier transformation frequency; HCl = hydrochloride.

**Brief user guide:** Step 1) Following the parent and output directories, the GUI requires input for the frame rate, pixel size, and file format. To increase analysis speed, frames can be skipped in an image series or videos with a very high frame rate (e.g., 30 f/s). Some microscopes require manual start and stop of video recordings. To standardize this data, there is the option to trim videos to one length (e.g., 20 s). Additionally, data can be overwritten if a secondary analysis is necessary. Step 2) Following data input, the first video is loaded into a preview (Supplementary Figure S1), and the script utilizes fluorescence intensity thresholds to detect the ROI for the heart. The heart can be rotated for ease of chamber selection. Step 3) User input determines the areas for atrium and ventricle segmentation by “drag-and-draw” to ensure correct detection, especially for dysmorphic phenotypes. Secondary fluorescence intensity thresholds separate chambers from the atrioventricular canal (AVC) to determine the area of each chamber for each frame. Within the ROI, fluorescence intensity changes are measured and fitted to a sine function and FFT using iterative approximation (Virtanen et al., 2020). The script generates a single output file for each image sequence and a collective spreadsheet containing all meta and quantitative data (Figure 2). Graphical results are displayed as GIF and PNG files. The applied look-up table (LUT) in the GIF displays differences in fluorescence intensity from minimum intensity (blue) to maximum intensity (yellow). Furthermore, the GIF displays the automated area selection throughout the whole video analysis. The PNG file shows the frequencies with the fitted sine function of each chamber (red dotted line) and an overlay of the raw data (blue and orange) (Figure 3C). The spreadsheet contains the complete quantitative data, including autocorrelation and fitting scores, which are metrics for data and fitting quality, respectively. A more detailed description of measurements and calculations of the parameters are included in the “Readme” of the tool (https://github.com/ToReinberger/pyHeart4Fish).

**Data interpretation guide:** Figure 3 shows the effects of two exemplary compounds on cardiac morphology and function compared with the control treatment (0.2% DMSO). These examples demonstrate that changes in contractility can differ between the atrium and ventricle, and such differences can indicate developmental toxicity (Figure 3B). Absolute contractility is defined as the area of the max. dilated chamber [µm^2^] (max. dilation) minus the area of max. contracted chamber [µm^2^] (max. contraction). To account for different heart sizes, the relative contractility [%] is calculated by dividing the absolute contractility by the area of max. dilated chamber. In particular, these examples show that an increased max. dilation combined with an increased area of max. contracted chamber does not necessarily result in contractility changes (Figure 3B opipramol diHCl; atrium). However, if the max. dilation is normal compared with controls but the area of the max. contracted chamber is vastly increased, the relative and absolute contractility is reduced and vice versa (Figure 3B opipramol diHCl, ventricle). Moreover, the absolute contractility can be reduced without the relative contractility significantly differing from DMSO controls (Figure 3B benzydamine HCl, atrium) due to the presence of smaller chambers. Figure 3C displays the heartbeat curves from the analysis of opipramol diHCl- or benzydamine HCl-treated zebrafish. The two compounds change chamber-specific contractility but do not induce arrhythmia (i.e., regular phase shift, arrhythmia score <0.7, and conduction score <0.5).

### 3.3 Quantitative heart phenotyping of hit compounds using *pyHeart4Fish*

To investigate the effects of the 13 hit compounds (Figure 1B) on heart morphology and function, we analyzed videos of fluorescent hearts with *pyHeart4Fish*. As all compounds were dissolved in DMSO, a 0.2% DMSO dilution was used as the negative control. More than 50% of identified hits were hydrochlorides (HCl). Therefore 20 µM sodium chloride (NaCl) was utilized as a salt control. As a positive control, we used 20 µM atorvastatin, a competitive 3-hydroxy-3-methylglutaryl-CoA (HMG-CoA) inhibitor and class D drug that is not recommended for use in early pregnancy (McIver and Siddique, 2022; TGA, 2022). In zebrafish, atorvastatin dose-dependently causes bradycardia, decreases the number of cardiomyocytes and causes a looping defect of the heart (Maerz et al., 2019). Another study showed that HMG-CoA inhibitors have strong adverse effects on pronephros development (Westhoff et al., 2020). In our study, all analyzed hits had effects on heart morphology and/or function in two zebrafish lines. If not otherwise indicated, all values (shown in brackets) were z-score normalized (Supplementary Table S2). A z-score smaller than −1.96 or greater than 1.96 displays statistical significance with an alpha error of 5%.

**FIGURE 4:**
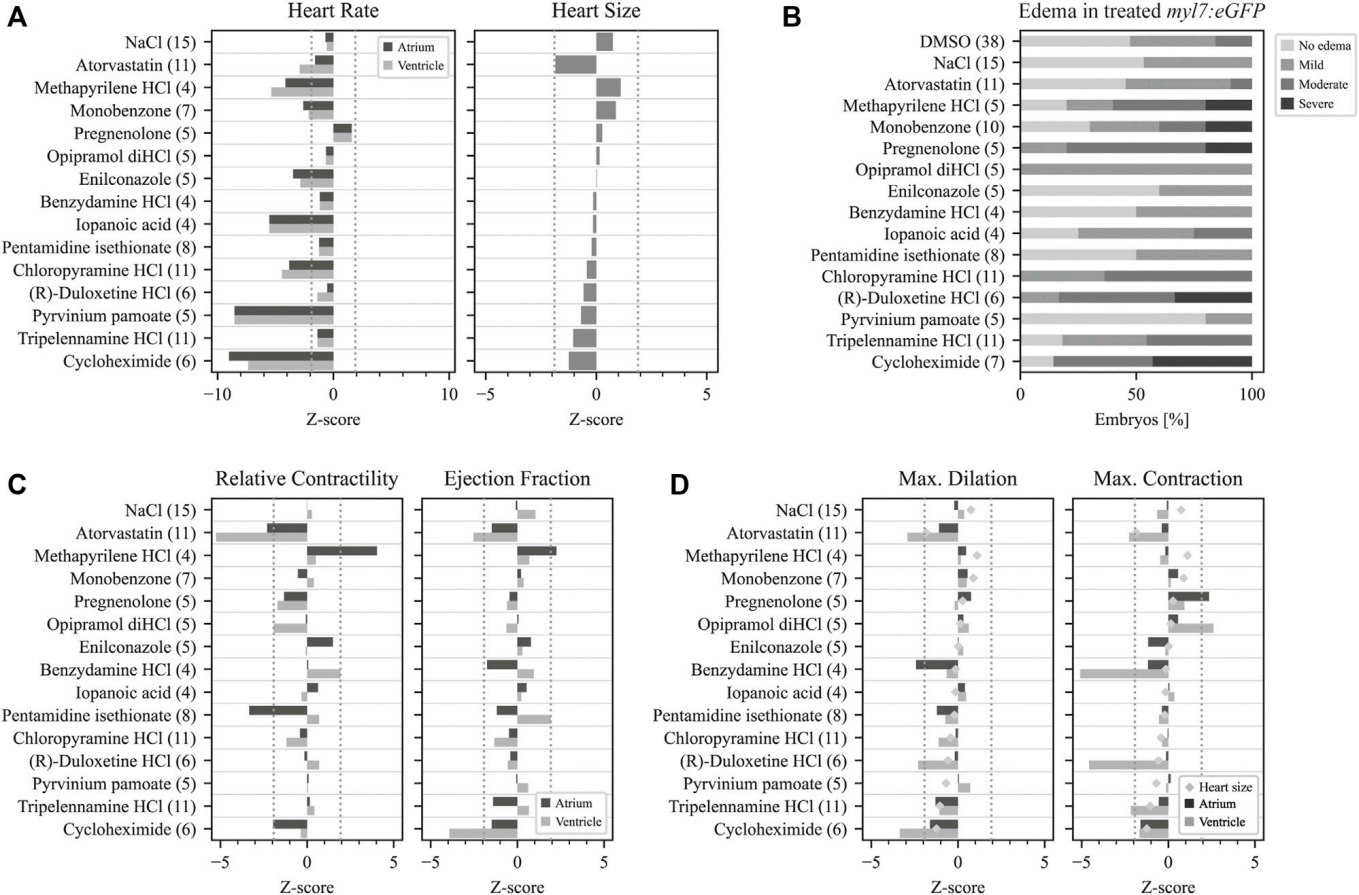
Quantitative data analysis with *pyHeart4Fish*. Analysis of treated fish with *pyHeart4Fish* revealed multiple functional heart phenotypes. **(A)** The left graph presents the detected heart rate derived from the fast fourier transformation (FFT). The right graph displays the approximate heart size in µm^2^. **(B)** Percentual data on the severity of manually assessed pericardial edema in treated Tg(*myl7:eGPF*)^twu34/+^ zebrafish. **(C)** The relative contractility derived from percentual size changes of each heart chamber (left graph). Approximate ejection fraction determined by chamber volume changes (right graph). **(D)** Mean max. heart chamber size (dilation) is presented on the left, while the mean min. heart chamber size (max. contraction) is displayed on the right. The number of fish analyzed is shown in brackets after the treatment condition. Dotted gray lines indicate z-scores of ±1.96 and statistical significance with an alpha error of 5%.

**FIGURE 5:**
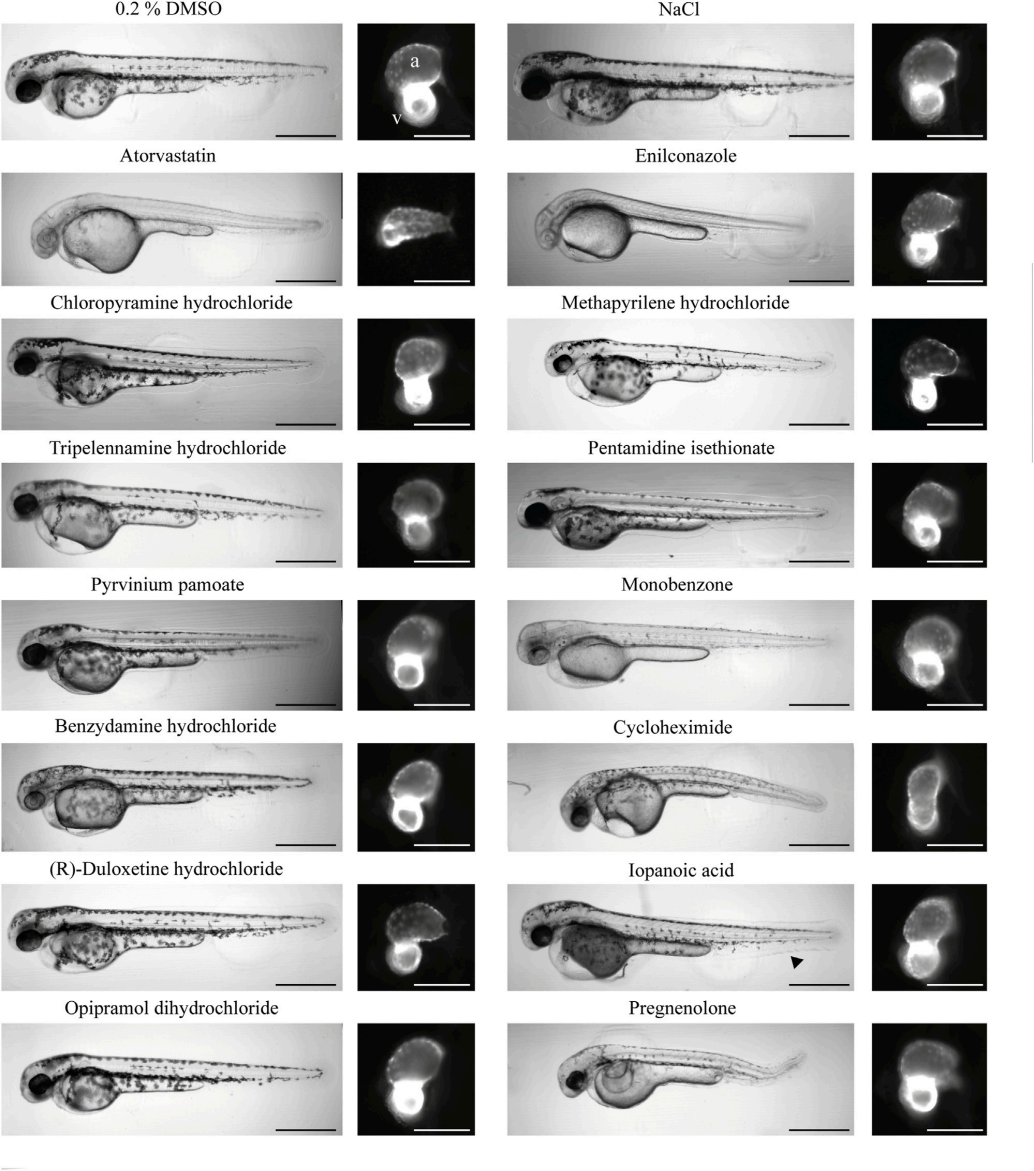
Morphology of treated zebrafish 2 dpf. An overview of the gross phenotypes of treated zebrafish 2 days post-fertilization. All compounds were dissolved in DMSO; therefore, DMSO served as the negative control. NaCl served as the salt control. The effects of atorvastatin treatment on zebrafish embryos were previously established; thus, this drug was used as a positive control for cardioactivity. The aquatic toxic compound enilconazole was used as the toxic positive control. Heart morphology was drastically affected only by enilconazole, cycloheximide, and pregnenolone. The black arrow in the image of iopanoic acid-treated zebrafish embryo indicates caudal fin deformity. The scale bar indicates 500 µm in overview brightfield images and 150 µm in fluorescence images. (a) Atrium; (v) ventricle.

**FIGURE 6:**
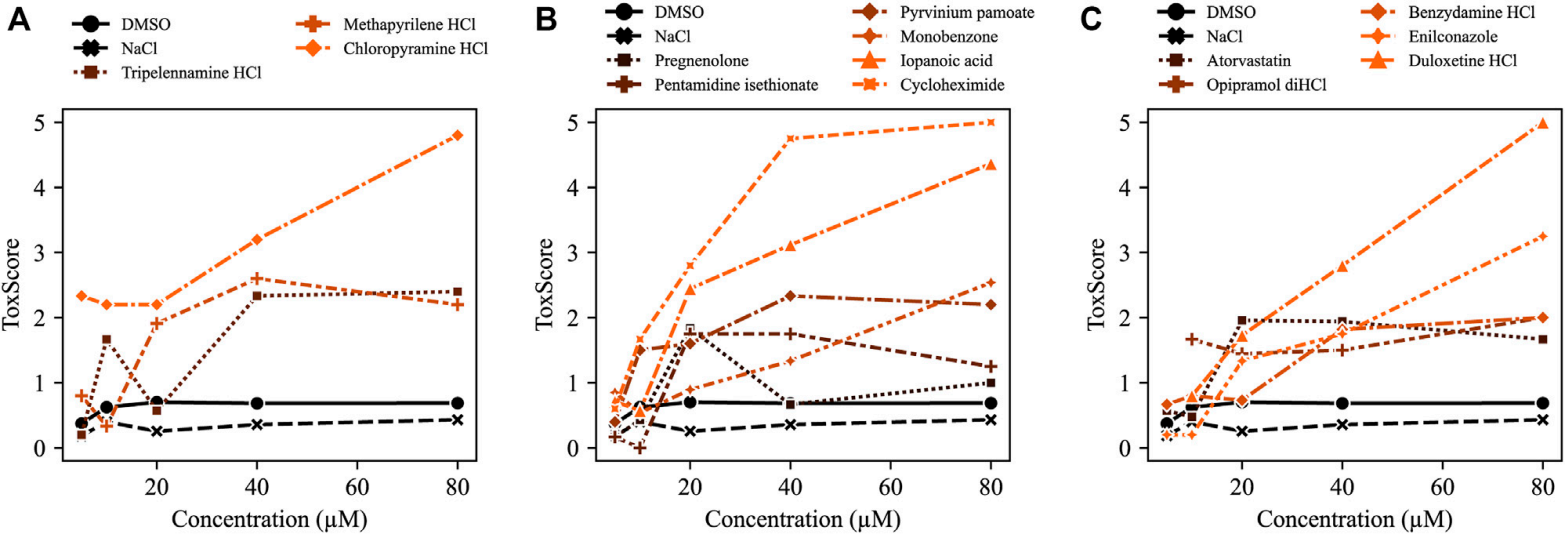
ToxScores of increasing compound concentrations. The compounds concentration-dependently increased ToxScores in treated zebrafish embryos. The ToxScores of **(A)** three HRH1 antagonists, **(B)** various compounds, and **(C)** hydrochlorides (HCls). The color gradient indicates the severity of the ToxScore from black (low ToxScore) to orange (high ToxScore).

Results from hits were compared with or normalized against 0.2% DMSO controls. The salt control NaCl (+0.2% DMSO) did not significantly affect heart morphology or functionality (Figure 4; Figure 5). Therefore, any effect of HCl on the heart can be attributed to the respective compound. In line with previously published studies, atorvastatin significantly decreased HR (Maerz et al., 2019) in the atrium (−2.90; n = 11) and the ventricle (−1.57). While atorvastatin did not cause electrophysiological conduction defects from the atrium to the ventricle, the difference in HR between chambers is likely caused by the AVC not correctly forming and looping not occurring during delayed development (Figure 5 and Supplementary Table S2). This AVC defect limits the ability of the tool to reliably detect chamber-specific HR. This developmental defect also explains the reduced chamber contractility, EF, and HS (Figures 4A,C). As a proof of concept, the aqua-toxic fungicide enilconazole, also known as imazalil, was tested (Huang et al., 2022). In both zebrafish lines, enilconazole caused developmental delays in 100% of the treated fish, inhibited pigmentation and significantly reduced HR (Figure 4A; 5). The toxic effects of enilconazole were dose-dependent (Figure 6B).

**Histamine receptor 1 (HRH1) antagonists:** Of the 134 compounds significantly altering HR, 15 compounds were HRH1 antagonists (Supplementary Table S6) and induced bradycardia. After the previously mentioned filters were applied, the three remaining HRH1 antagonists were chloropyramine HCl (CHCl), methapyrilene HCl (MP) and tripelennamine HCl (TRP). These compounds are first-generation HRH1 antagonists and belong to the class of ethylene diamines (Shawky and Seifeldin, 2015). All three antagonists decreased HR in zebrafish compared with DMSO controls (Figure 4A). CHCl and MP induced a slower HR in the ventricle than in the atrium, indicating a conduction defect. At 20 μM, 50% of MP- and 18.2% CHCl-treated embryos displayed a Mobitz type II atrioventricular block (AVB). Relative contractility was significantly increased in the atrium of MP-treated embryos (4.03; n = 4), while the ventricle was unaffected (0.50). TRP-treated embryos tended to have smaller hearts than the DMSO controls (−1.04) (Figure 4A); however, development was not delayed (Figure 5). Max. contraction significantly increased in the ventricle of TRP-treated fish (−2.12) (Figure 4D). Most zebrafish treated with HRH1 antagonists displayed mild to moderate pericardial edema (Figure 4B, 5). Further, the ToxScores of MP and TRP did not exceed 2.6, while 80 µM CHCl induced cardiac arrest in all zebrafish embryos, leading to a ToxScore of 4.8 (Figure 6A).

**Cycloheximide (CHX):** CHX is an antibiotic and a prokaryote 80s ribosome inhibitor. Treatment with CHX drastically decreased HR (Atrium: −9.02; Ventricle: −8.87, n = 6) in both zebrafish lines (Figure 4A and Supplementary Figure S2). The relative contractility of the ventricle was not affected (−0.36) while atrium contractility significantly decreased compared with DMSO controls (−1.96) (Figure 4C). Overall HS was smaller than average (−1.24) and development was delayed (Figure 5), explaining the reduced max. contraction and max. dilation in the atrium and ventricle (Figure 4D). The ventricle max. dilation (−3.36) and max. contraction (−1.66) were significantly reduced, and this data was confirmed by a reduction in EF (−3.93) (Figure 3C). Furthermore, most CHX-treated embryos displayed moderate to severe pericardial edema and bent tails (Figure 4B, 5). The detrimental effects of CHX were also reflected in the calculated ToxScore of CHX; doses higher than 40 µM induced cardiac arrest and severe pericardial edema (Figure 6B).

**Pyrvinium pamoate (PP):** The antifungal and anthelmintic PP, approved by the FDA (Li et al., 2022), was investigated as an anti-cancer drug and found to act *via* multiple pathways such as WNT signaling, inhibition of mitochondrial NADH-fumarate, tumor stemness, the unfolded protein response and hedgehog signaling (Schultz and Nevler, 2022). In our screen, PP caused a significant decrease in HR in both heart chambers (−8.54; n = 5). However, the treatment did not affect relative contractility, conduction, and HS (Figure 4). Heart morphology was normal, and no teratogenic effects of PP at 20 µM were detected (Figure 5).

**Pentamidine isethionate (PEN):** The antiprotozoal compound PEN interacts with protozoan AT-rich DNA regions. In zebrafish embryos at 2 dpf, PEN caused bradycardia in the atrium and ventricle (−1.24; n = 8) (Figure 4A). While overall HS did not differ from the controls (−0.21), PEN significantly decreased the relative contractility of the atrium (−3.86) (Figures 4A,C). While the max. dilation was smaller (−1.22), the max. contraction did not differ from the controls (−0.37) (Figure 4D). A direct comparison of the heartbeat videos confirmed the atrium’s reduced contractility in the presence of PEN (Supplementary Figure S3). However, the gross morphology of the embryos did not reveal teratogenic effects (Figure 5).

**Monobenzone (MBEH):** MBEH is a monobenzyl ether of hydroquinone that induces depigmentation (Oliver et al., 1939). As shown in Figure 5, MBEH caused depigmentation without delaying development in zebrafish embryos and did not affect their heart morphology at 2 dpf (Figure 5). Analysis of the heart showed significant bradycardia in both heart chambers (atrium: −2.60, ventricle: −2.12; n = 7) (Figure 4A). Exposure to MBEH did not cause any other alterations to heart phenotypes. Further, MBEH treatment did not exceed a ToxScore of 2.5 at 80 μM; this score was caused by developmental delay, reduced HR and mild pericardial edema (Figure 6B; Supplementary Figure S7A).

**Benzydamine HCl (BZY):** The non-steroidal anti-inflammatory drug (NSAID) BZY inhibits phospholipase A_2_ activity by preventing arachidonic acid release from phospholipids (Turnbull, 1995; Passali et al., 2022). Additionally, it weakly inhibits prostaglandin endoperoxide H synthase 1 and 2 by decreasing prostaglandin production (Blackwell et al., 1975; Modéer and Yucel-Lindberg, 1999; Krzeszowski et al., 2015). In this drug screen, BZY caused mild bradycardia (−1.16; n = 4) and decreased the max. dilation of the atrium (−2.90), thereby decreasing atrial EF (−1.75). The ventricle dilated normally; however, it contracted significantly more than the controls (−5.07) (Figure 3B and 4C-D). Approximated HS was normal, and no conduction defects or arrhythmias were detected (Figure 4C and Supplementary Figure S4). BZY caused pericardial edema in 50% of the treated zebrafish embryos (Figure 4B).

**(R)-Duloxetine HCl (DLX):** The serotoninergic 5-HT-norepinephrine reuptake inhibitor (SNRI) DLX blocks norepinephrine and serotonin transporters (Park et al., 2020; Tran et al., 2021). The mean HR in *myl7:eGFP* zebrafish embryos was not significantly decreased by DLX; however, articular and ventricular HR was different (atrium: −0.54, ventricle: −1.38; n = 6). Indeed, manual analysis of the heartbeat videos revealed that one-third of the embryos displayed atrial flutter (Fisher’s exact test *p* = 0.19) (Supplementary Figure S5A), which could also be detected using the BF module of *pyHeart4Fish* (Supplementary Figure S5B). Additionally, exposure to DLX caused mild (33%) to severe (33%) pericardial edema in zebrafish embryos (Figure 4B). However, the ranked Spearman test did not show a correlation between atrial flutter and edema severity (data not shown; r = 0.35). Nevertheless, DLX dose-dependently increased the ToxScore, resulting in complete lethality at 80 µM (Figure 6C).

**Opipramol diHCl (OPI):** OPI was developed originally as an antidepressant and later used as a treatment for somatoform disorder (Gahr et al., 2017). The sigma receptor agonist did not affect HR in *myl7:eGFP* or change HS (Figure 4A). On the other hand, the max. area of the contracted ventricle was significantly increased by OPI (2.61) (Figure 3B, 4D). As shown in Figure 4B and Figure 5, OPI caused mild edema and induced a mild developmental delay.

**Iopanoic acid (IOP):** The contrast agent IOP caused significant bradycardia (−5.53; n = 4) with regular conduction. Contractility, HS and EF were not affected. Non-etheless, IOP treatment caused developmental delay and mild edema in *myl7:eGFP* zebrafish and moderate edema in *fli1a:eGFP* zebrafish. Furthermore, we observed similar caudal fin deformities in several fish, as indicated by the arrow in Figure 5. The number of developmental delays, and the severity of pericardial edemas increased with increasing IOP doses, and HR decreased, leading to a ToxScore of 4.4 at 80 μM, with most embryos dying of cardiac arrest (Figure 6B).

**Pregnenolone (P5):** P5 is a gonadal steroid hormone and adrenal corticosteroid precursor, therefore it is often referred to as a prohormone. In *myl7:eGFP,* zebrafish P5 caused mild tachycardia (1.58, n = 5) (Figure 4A). The relative contractility of both heart chambers was affected by impaired contraction. However, HS and morphology were not affected. P5 exposure for 24 h led to teratogenic effects on the embryos. All embryos were smaller than controls and displayed moderate to severe pericardial and yolk edema (Figure 4B), developmental delays, reduced skin pigmentation and bent tails (Figure 5).

## 4 Discussion

Increasing the current knowledge base of prenatal and postnatal drug safety is of utmost importance, as teratogenic drugs could have debilitating effects on a person’s life. However, human drug toxicity information is often based on retrospective data. Since 1993, strict regulations mandate that embryo-fetal developmental and reproductive toxicity (DART) studies are performed in two animal models: rodents and non-rodents (Barrow, 2018). However, DART studies are labor- and cost-intensive and require large numbers of animals. Therefore, an increasing number of large-scale drug screens are performed in zebrafish as an alternative animal model (Piersma, 2004; Asnani and Peterson, 2014; Cully, 2019; Zhao et al., 2019; Patton et al., 2021).

In this study, we conducted a high-content screen for cardioactive compounds in zebrafish and robustly analyzed heart morphology and function with our newly-developed Python-based tool, *pyHeart4Fish*. Images and videos were acquired from embryos arrayed in 96-well plates to capture consistent lateral and ventral views of over 10,000 embryos. Standardized datasets allowed for semi-automated analysis of quantitative heart phenotypes. Validation was performed with a second transgenic zebrafish line and additional controls to strengthen data validity. The subset of hits chosen for this study confirmed existing data and revealed new insights into the functional cardiac effects of 13 compounds, of which eight induced significant bradycardia. Our screen did not only mirror the cardiac effects and/or side effects from cardioactive compounds but also revealed changes in other phenotypes, such as depigmentation and developmental toxicity, as exemplified by the effects of enilconazole. Our findings match a report from Huan *et al.* in 2022, which showed a significantly lower HR in enilconazole-treated embryos. However, they did not report reduced pigmentation and developmental delays (Huang et al., 2022). Hence, our screening approach with subsequent analysis using *pyHeart4Fish* is sensitive enough to replicate known effects and may provide new insights for use in future drug screens.

Within the filtered subset, three HRH1 antagonists (CHCl, MP and TRP) induced bradycardia in zebrafish embryos at 2 dpf (Figure 4A). Chamber-specific HR data combined with the AVB score revealed CHCl and MP caused Mobitz type II AVB. AVBs caused by HRH1 impairment were previously reported in frogs, guinea pigs, rabbits, dogs and humans (Kar et al., 2012; Einis, 2013). These findings concur with those of a study reporting that disrupted HRH1 signaling represses cardiac differentiation and maturation in human pluripotent stem cells (Zhu et al., 2020). Even though studies on antihistamine treatments in pregnant women show conflicting results, the current consensus is that HRH1 antagonists do not cause CHDs in humans (Shawky and Seifeldin, 2015; Etwel et al., 2017).

Our study revealed 10 additional compounds that directly affect cardiac development and function. The toxic protein translation inhibitor CHX was initially used to control mildew and short blight in onions and larch (Müller et al., 2011). However, during its use, it was known to be highly phytotoxic against certain plants, such as peas, and is no longer available as a fungicide (Müller et al., 2011; Gupta, 2017). CHX caused skeletal defects and dactyly defects in mice, impaired bovine oocyte development *in vitro* and developmental delay in early-stage rainbow trout (*Oncorhynchus mykiss*) embryos (Lary and Hood, 1982; Nagler, 2000; Hashimoto et al., 2003; German et al., 2015). In 2013 Yang *et al.* reported that 71 μM CHX decreased ventricular volume in zebrafish embryos (Yang et al., 2014). However, they did not report any data on HR or atrium volume changes. Here, we found that 20 μM CHX induced developmental delay and pericardial edema accompanied by bradycardia, reduced atrial contractility, and overall decreases in HS and EF. Our data indicate that CHX has strong cardiotoxic effects; this outcome aligns with recent findings showing that CHX induces apoptosis in cardiomyocytes (Zuo et al., 2018; Li et al., 2020).

In a mouse myocardial infarction model, the antifungal agent PP improves cardiac contractility without cardiac rupture (Murakoshi et al., 2013). In our zebrafish study, PP induced significant bradycardia but did not affect cardiac function or morphology, including contractility. While PP acts on multiple pathways and has been extensively studied in different models, to our knowledge, we are the first to report significant bradycardia after PP exposure in zebrafish.

PEN is classified as a B3 drug, as exposure in different animal models leads to long QT syndrome, model-dependent bradycardia or tachycardia, and sudden cardiac arrest (Milan et al., 2003; Sanguinetti and Tristani-Firouzi, 2006; Dennis et al., 2012; TGA, 2022). These side effects are caused by off-target blockage of the potassium I_Kr_/hERG current (Sanguinetti and Tristani-Firouzi, 2006; Dennis et al., 2012). In this study, exposure to PEN caused mild bradycardia with reduced atrial contractility. Although PEN did not have toxic effects on gross developmental morphology, it affected heart function.

The depigmentation agent MBEH causes spreading contact vitiligo by selectively destroying melanocytes and inducing an immune reaction against melanocytes (Boissy and Manga, 2004; van den Boorn et al., 2010). Additionally, data from Boisson et al. suggests that the conversion of phenolic or catecholic derivatives, such as MBEH, can induce oxidative stress (Boissy and Manga, 2004). In our screen, MBEH induced depigmentation without inducing developmental toxicity. Moreover, MBEH treatment caused bradycardia in zebrafish embryos without affecting morphology or function. To our knowledge, this is the first study to report bradycardia caused by MBEH exposure in zebrafish.

BZY is an NSAID, one of the most commonly prescribed group of drugs used to treat acute and chronic pain during pregnancy. In a case-control study from 2012, 22.6% of women reported using NSAIDs in the first trimester of pregnancy (Hernandez et al., 2012). NSAIDs are also administered to close patent *ductus arteriosus* in preterm neonates (Westhoff et al., 2020). In 2015, three cases of premature constriction of the fetal *ductus arteriosus* after maternal self-medication with BZY during pregnancy were reported (Krzeszowski et al., 2015). Premature *ductus arteriosus* is associated with an increased risk of neonatal pulmonary hypertension (Zenker et al., 1998; Chen et al., 2022). In patients, pulmonary hypertension leads to an enlarged ventricle that has to pump harder to move the blood through narrowed or blocked pulmonary arteries (Vonk Noordegraaf et al., 2017). We found that exposure of zebrafish embryos to BZY impaired atrial dilation and thereby decreased atrial EF. Moreover, the relative ventricle contractility increased significantly, probably to compensate for impaired atrial EF. Zebrafish do not have a *ductus arteriosus* or pulmonary circulation, instead they have a unique aortic and pharyngeal arch artery (Nakajima et al., 2021). In zebrafish, the ventricle drives blood circulation (Gu et al., 2017; González-Rosa, 2022; Trinidad et al., 2022), so increased pressure caused by an impaired pharyngeal arch artery would also impact the ventricle. Results from other zebrafish drug screens indicate that NSAIDs cause severe renal malformations, implying that BZY is embryotoxic (Westhoff et al., 2020). While several studies imply that NSAIDs have developmental toxicity, the overall risk depends on the treatment concentration, duration and gestational stage; therefore, BZY is classified as a B2 risk compound (Nielsen et al., 2001; Hernandez et al., 2012; Dathe et al., 2022; TGA, 2022).

Serotonin reuptake inhibitors (SRIs) are taken in 2%–6% of all pregnancies to treat anxiety, depression, phobias and other disorders (Ornoy and Koren, 2019). DLX was the most commonly prescribed SNRIs for pain control and mood disorders in the U.S. in 2017 (Goldstein et al., 2004; Agency for Healthcare Research and Quality, 2017; Park et al., 2020). According to the Toxicology Investigators Consortium, SNRIs caused 10.6% of antidepressant-related toxic events in 2021 (Love et al., 2022). Data on the treatment outcomes of DLX during pregnancy is controversial, yet several studies demonstrate that it causes congenital cardiovascular malformations in neonates (Diav-Citrin et al., 2008; Merlob et al., 2009; Jimenez-Solem et al., 2012; Knudsen et al., 2014; Ornoy and Koren, 2019; Park et al., 2020). Here, we found DLX induces pericardial edema and atrial flutter in 33% of treated embryos, although the two events were not correlated. Apart from single reports from extensive human studies, our study is the first demonstration of DLX-induced atrial flutter in zebrafish.

OPI is a tricyclic antidepressant (TCA) that is structurally similar to SRIs, but with potentially fewer side effects (Müller et al., 2004). There is little information about OPI-associated embryonic developmental risks. However, several studies show no significant association between TCA treatment and congenital malformation (Simon et al., 2002; Davis et al., 2011). In contrast to the common perception that TCAs are safer than SRIs, some studies demonstrate that TCAs carry a greater risk of developmental delays and preterm birth (Reis and Källén, 2010; Rommel et al., 2022). A small double-blind trial from 2002 tested OPI as a premedication in anesthesiology and found OPI did not affect patients’ HR and blood pressure (Gerlach et al., 2002). That study is in agreement with our data showing that zebrafish HR was unaffected by OPI. Of note, OPItreatment in zebrafish leads to significantly reduced relative ventricular contractility compared to DMSO controls. Therefore, our findings point toward risks for prenatal OPI prescription.

Literature about the deiodinase inhibitor IOP demonstrates the drug has no effects on cardiac *β*-adrenoceptor density and HR (Stäubli and Studer, 1986; Perret et al., 1992). In 2007, a study even showed IOP had cardioprotective properties during regional anesthesia (Berti et al., 2007). However, IOP accumulates in the heart of guinea pigs, and overdose induces bradycardia (Lindenmeyer et al., 1984). Therefore, the significant bradycardia observed in previous studies, together with the observed edema in zebrafish embryos and the dose-dependent increase in the ToxScore, point toward IOP having embryotoxicity.

It is well known that prenatal hormone exposure is associated with developmental and metabolic defects (Lindenmeyer et al., 1984; Hsu et al., 2006). Prohormone P5 is one of the few compounds within the drug library that is not approved. In zebrafish, P5 promotes cell migration during epiboly (∼five to nine hpf), a process in which embryonic cells spread from the animal pole to the opposite pole to enclose the yolk (Hsu et al., 2006; Kolas et al., 2022). However, to our knowledge, there are no studies of P5 treatment conducted in zebrafish embryos or 24–48 hpf; thus we report P5-induced teratogenicity and dose-dependent tachycardia for the first time (Supplementary Figure S7A).

The high-content screening approach of this study revealed detailed cardiac effects of various approved and over-the-counter drugs on the developing embryo. The wide therapeutic classes of drugs available and the vastly different chemical structures demonstrate the necessity of detailed drug testing for embryotoxicity, especially for cardiac phenotypes. Contractility phenotypes and arrhythmias in early life could weaken the developing heart and lead to CHD and early death (Ran et al., 2022).

A limitation of our screen is that we only identified cardiotoxic effects at one compound concentration (20 µM), thereby not detecting concentration-dependent drug activity. A more detailed analysis of hit compounds is currently being performed in our laboratory. Another limitation of our study is that drug exposure occurred *via* water and this method potentially impaired drug bioavailability. Although we removed the chorion barrier of the embryos using chemical dechorionation, the transdermal uptake of compounds may vary depending on their chemical properties. A current limitation of our tool is the requirement for fluorescent-labeled hearts; nevertheless, a version of the tool for HR analysis of BF zebrafish videos is provided. Currently, user-based determination of the atrium and ventricle in *pyHeart4Fish* can improve the overall readout, although an underdeveloped AVC or malformed heart may result in erroneous data.

In conclusion, we demonstrated that our novel screening tool based on the 3 R principle, *pyHeart4Fish*, not only confirmed findings from the current literature but also provided insights into the toxicity of 13 cardioactive compounds. We provide new insights into drug-induced, chamber-specific anomalies in cardiac function, e.g., indications of DLX-induced atrial flutter and MP-induced AVB. With *pyHeart4Fish,* we provide a novel resource for comprehensive and objective semi-automated heart analysis in fish that drastically increases the data readout while saving time on analysis, further contributing to reducing animal use. In general, using *pyHeart4Fish* can improve our understanding of CVDs and cardiotoxic effects to push forward the development of effective therapeutics.

Finally, this study may serve as a basis for further investigation of the pre- and postnatal toxicity of approved compounds. Many drugs were approved long before DART studies became a requirement, so such drugs might have a high risk for adverse effects during pregnancy for the mother and child. Therefore, more detailed screens of approved drugs must be performed to provide a resource for medical professionals to consult before drugs are prescribed and used.

## Data Availability

The raw data supporting the conclusion of this article will be made available by the authors, without undue reservation.

## Supplementary material

pyHeart4FIsh is available via Github https://github.com/ToReinberger/pyHeart4Fish.
