# Supplementary material for: pyHeart4Fish: Chamber-specific heart phenotype quantification of zebrafish in high-content screens
Source: Front Cell Dev Biol. 2023 Apr 11;11:1143852. doi: 10.3389/fcell.2023.1143852 (PMC10126419; doi:10.3389/fcell.2023.1143852)
Supplement: Supplementary file 3 [file Presentation1.PPTX]

## Slide 1
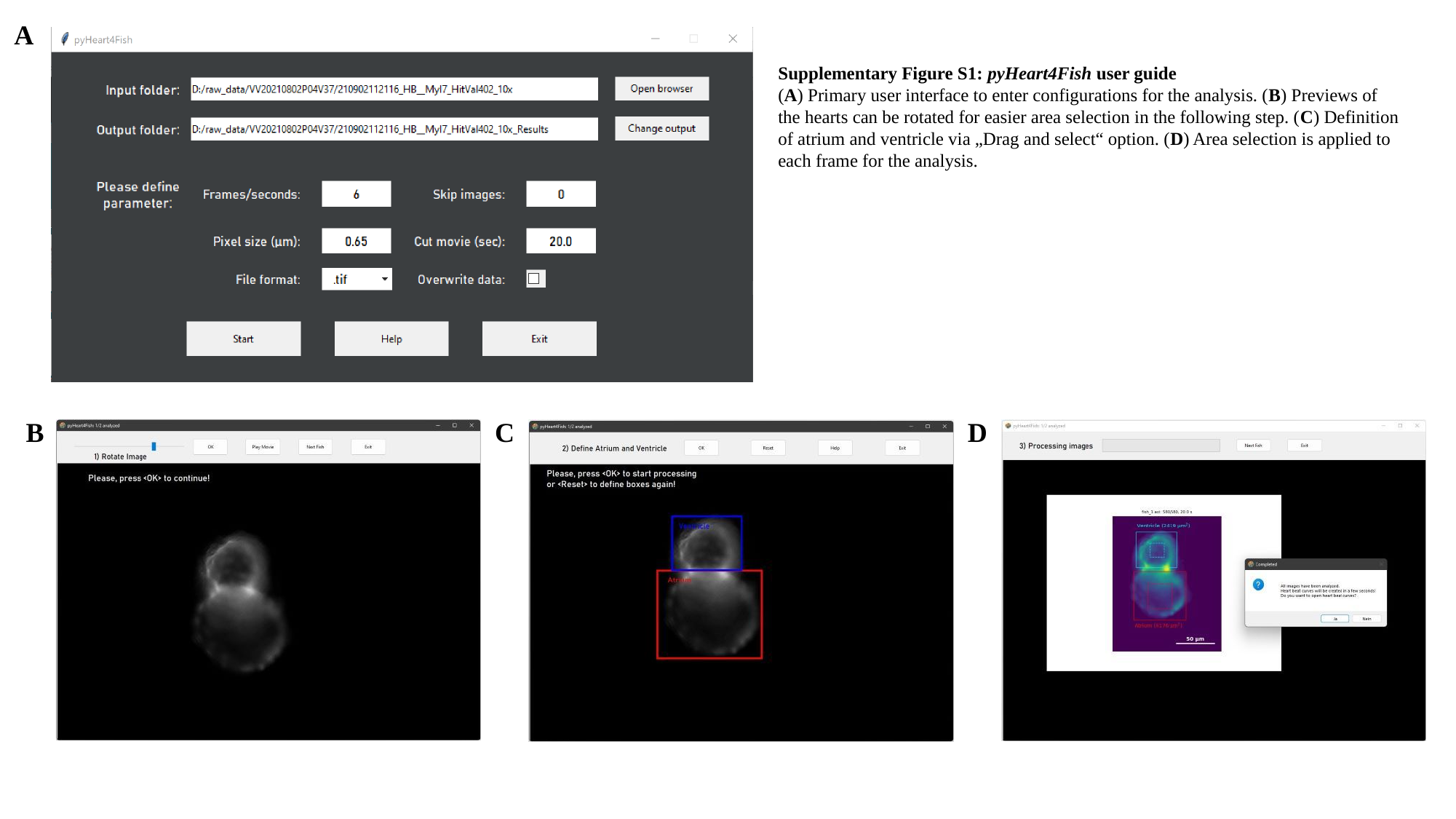

A
Supplementary Figure S1: pyHeart4Fish user guide
(A) Primary user interface to enter configurations for the analysis. (B) Previews of the hearts can be rotated for easier area selection in the following step. (C) Definition of atrium and ventricle via „Drag and select“ option. (D) Area selection is applied to each frame for the analysis.
B
C
D

## Slide 2
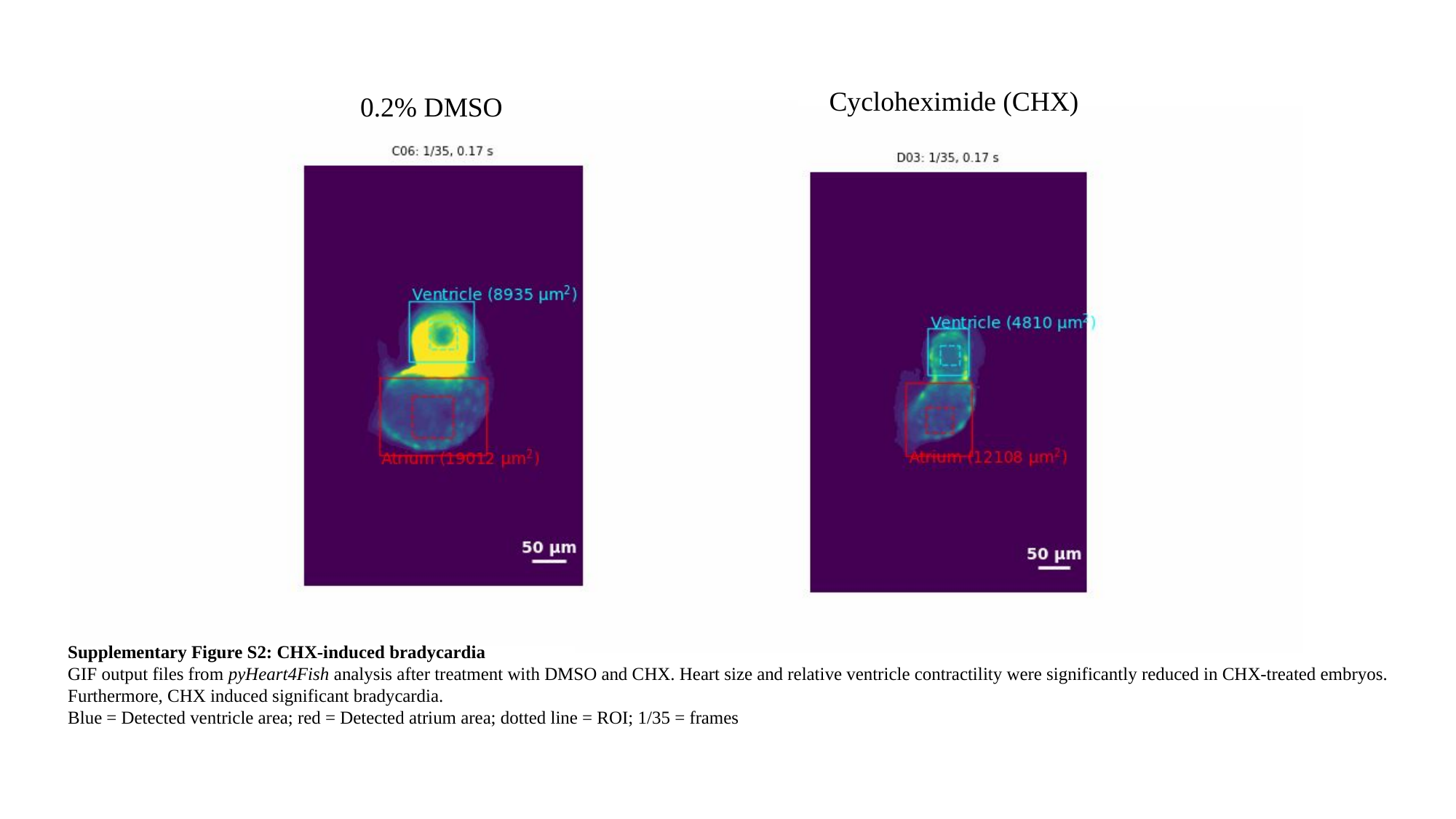

Cycloheximide (CHX)
0.2% DMSO
Supplementary Figure S2: CHX-induced bradycardia
GIF output files from pyHeart4Fish analysis after treatment with DMSO and CHX. Heart size and relative ventricle contractility were significantly reduced in CHX-treated embryos. Furthermore, CHX induced significant bradycardia.
Blue = Detected ventricle area; red = Detected atrium area; dotted line = ROI; 1/35 = frames

## Slide 3
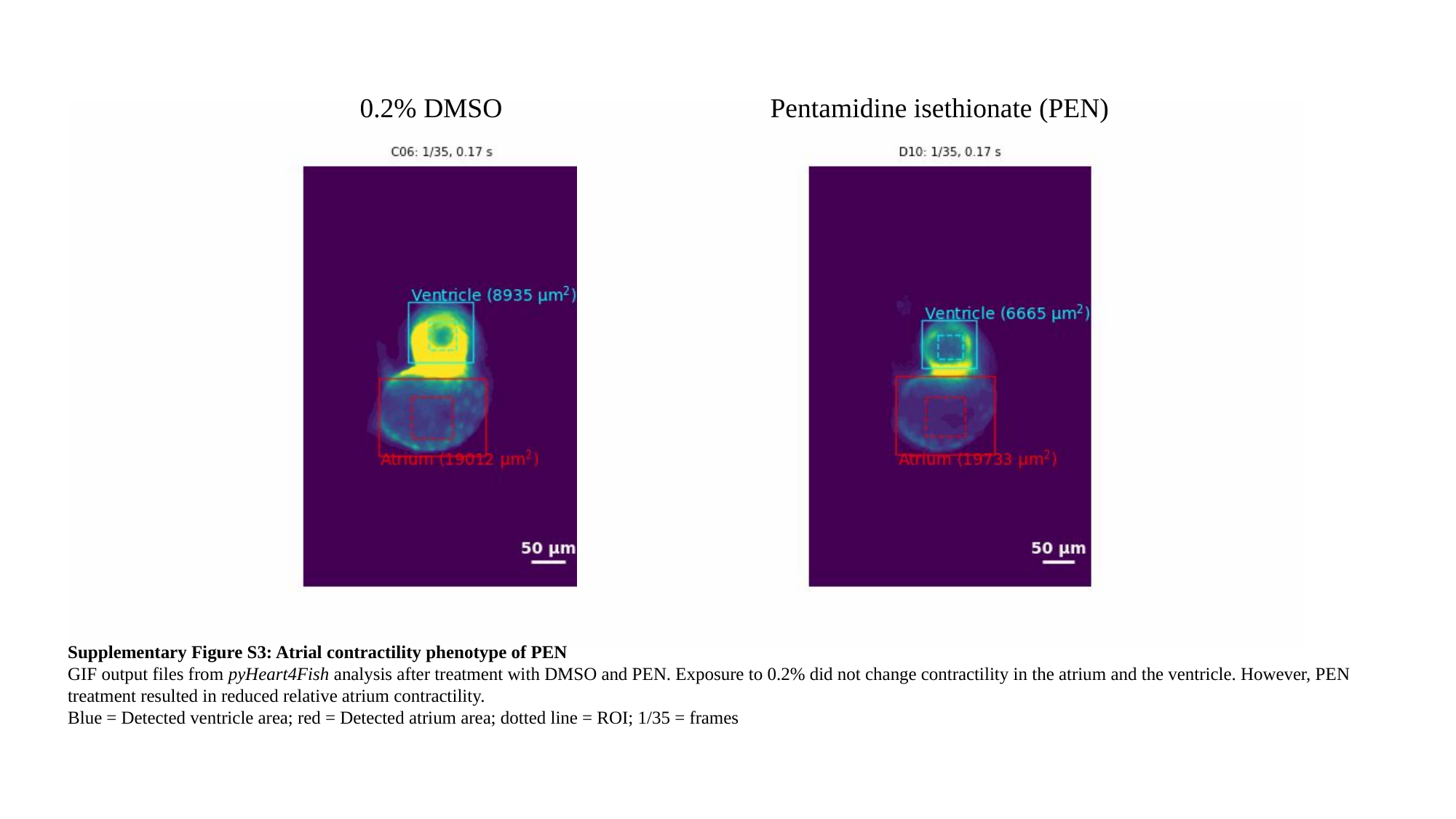

0.2% DMSO
Pentamidine isethionate (PEN)
Supplementary Figure S3: Atrial contractility phenotype of PEN
GIF output files from pyHeart4Fish analysis after treatment with DMSO and PEN. Exposure to 0.2% did not change contractility in the atrium and the ventricle. However, PEN treatment resulted in reduced relative atrium contractility.
Blue = Detected ventricle area; red = Detected atrium area; dotted line = ROI; 1/35 = frames

## Slide 4
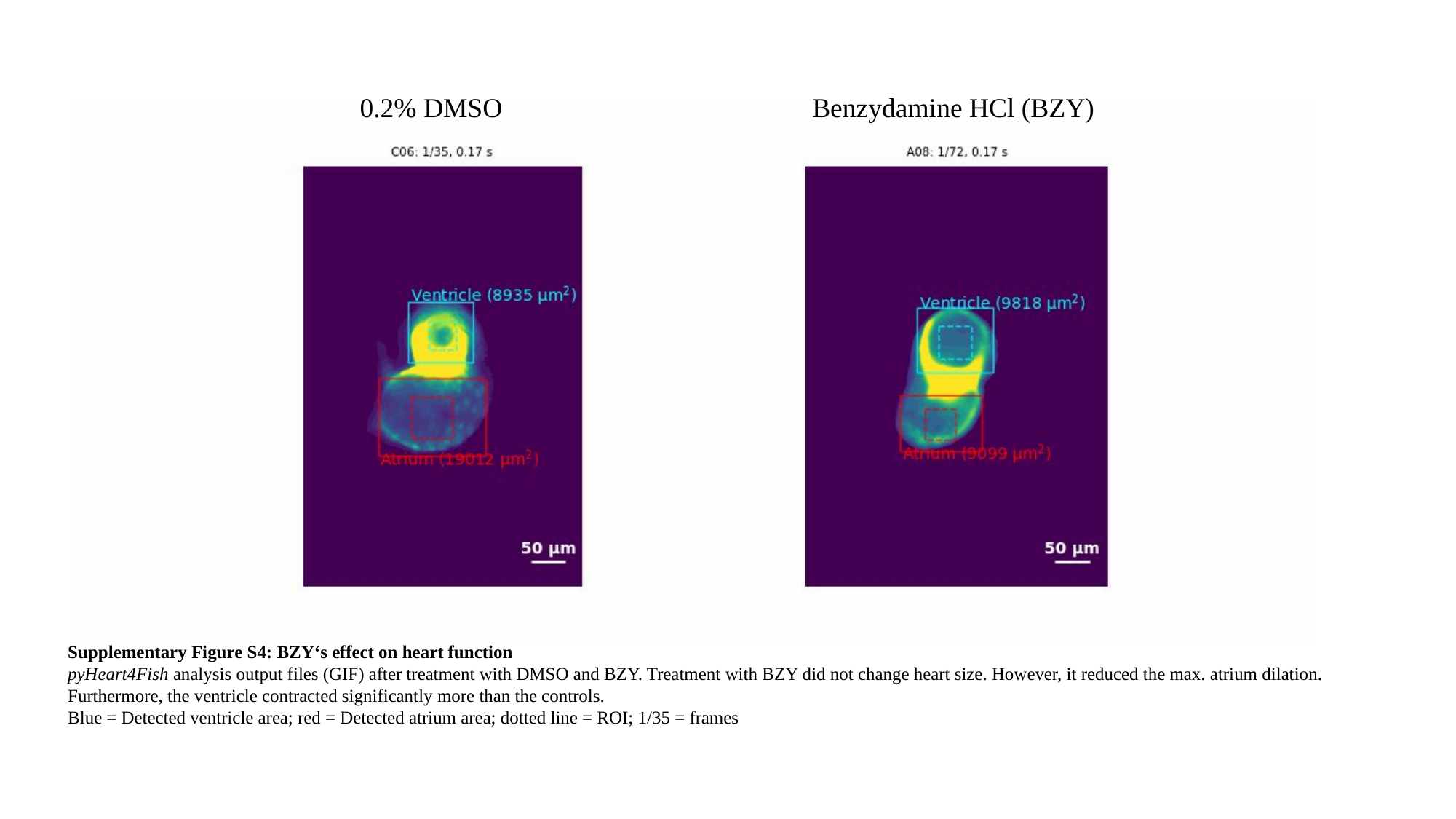

Benzydamine HCl (BZY)
0.2% DMSO
Supplementary Figure S4: BZY‘s effect on heart function
pyHeart4Fish analysis output files (GIF) after treatment with DMSO and BZY. Treatment with BZY did not change heart size. However, it reduced the max. atrium dilation. Furthermore, the ventricle contracted significantly more than the controls.
Blue = Detected ventricle area; red = Detected atrium area; dotted line = ROI; 1/35 = frames

## Slide 5
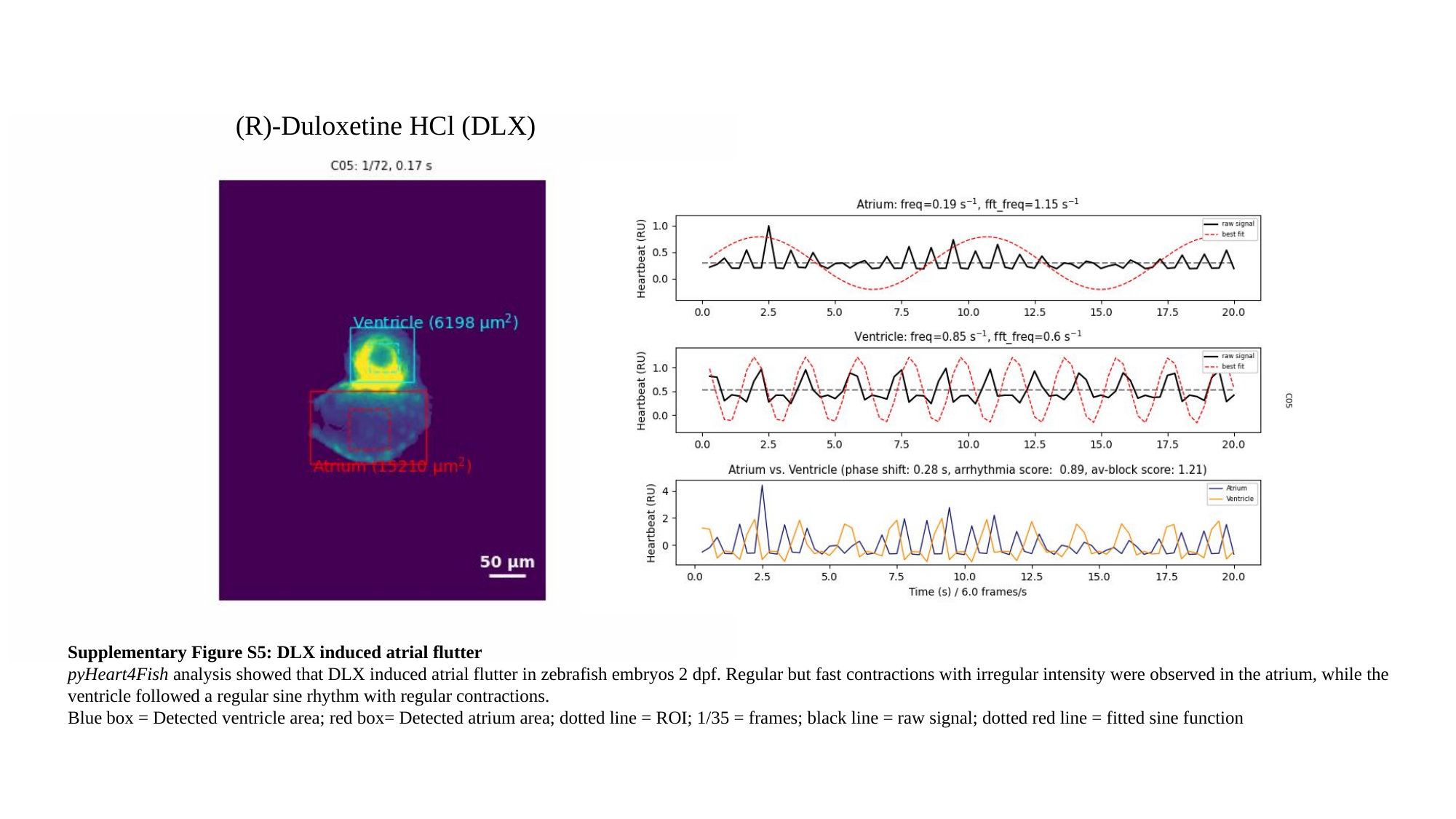

(R)-Duloxetine HCl (DLX)
Supplementary Figure S5: DLX induced atrial flutter
pyHeart4Fish analysis showed that DLX induced atrial flutter in zebrafish embryos 2 dpf. Regular but fast contractions with irregular intensity were observed in the atrium, while the ventricle followed a regular sine rhythm with regular contractions.
Blue box = Detected ventricle area; red box= Detected atrium area; dotted line = ROI; 1/35 = frames; black line = raw signal; dotted red line = fitted sine function

## Slide 6
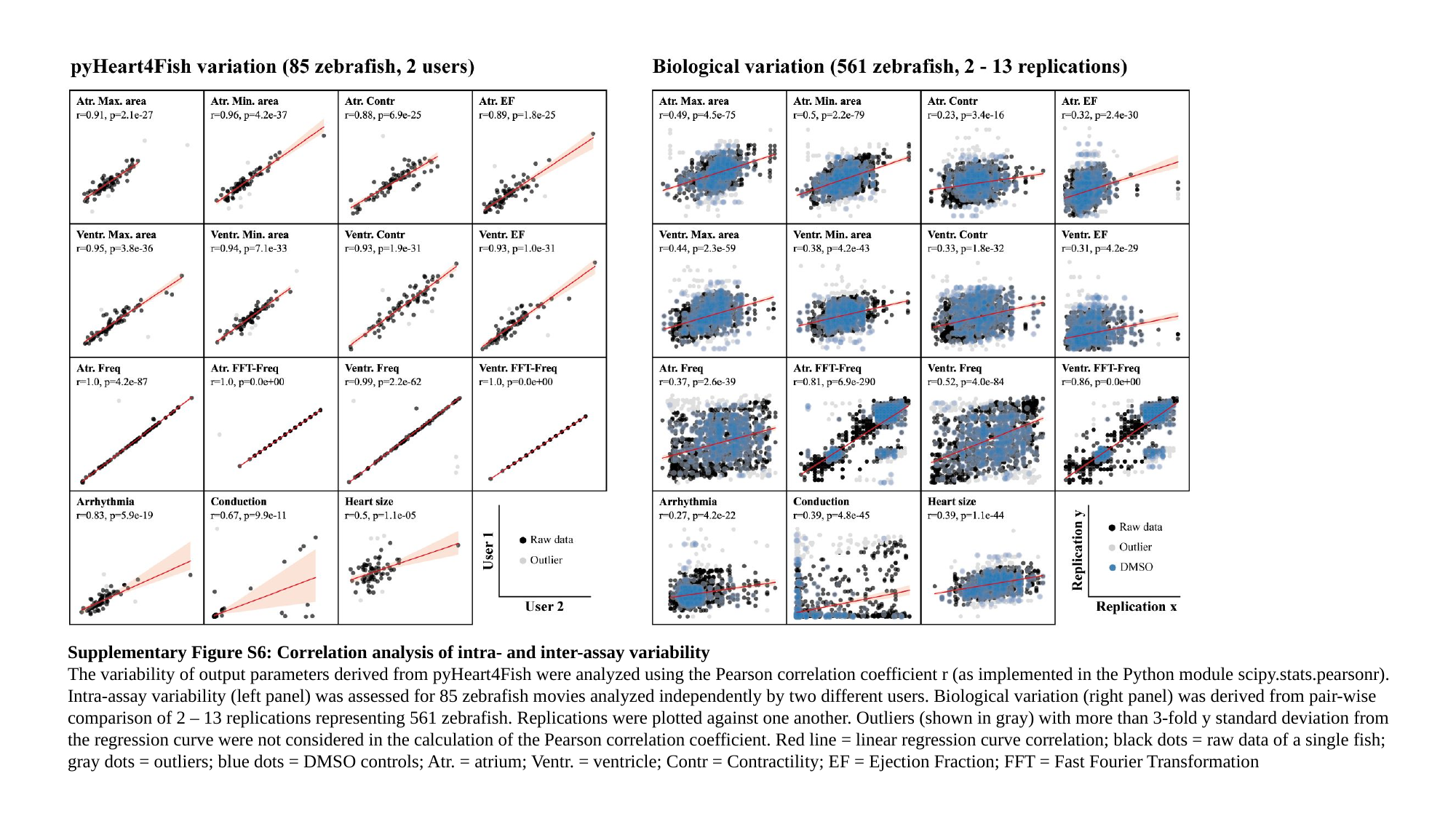

Supplementary Figure S6: Correlation analysis of intra- and inter-assay variability
The variability of output parameters derived from pyHeart4Fish were analyzed using the Pearson correlation coefficient r (as implemented in the Python module scipy.stats.pearsonr). Intra-assay variability (left panel) was assessed for 85 zebrafish movies analyzed independently by two different users. Biological variation (right panel) was derived from pair-wise comparison of 2 – 13 replications representing 561 zebrafish. Replications were plotted against one another. Outliers (shown in gray) with more than 3-fold y standard deviation from the regression curve were not considered in the calculation of the Pearson correlation coefficient. Red line = linear regression curve correlation; black dots = raw data of a single fish; gray dots = outliers; blue dots = DMSO controls; Atr. = atrium; Ventr. = ventricle; Contr = Contractility; EF = Ejection Fraction; FFT = Fast Fourier Transformation

## Slide 7
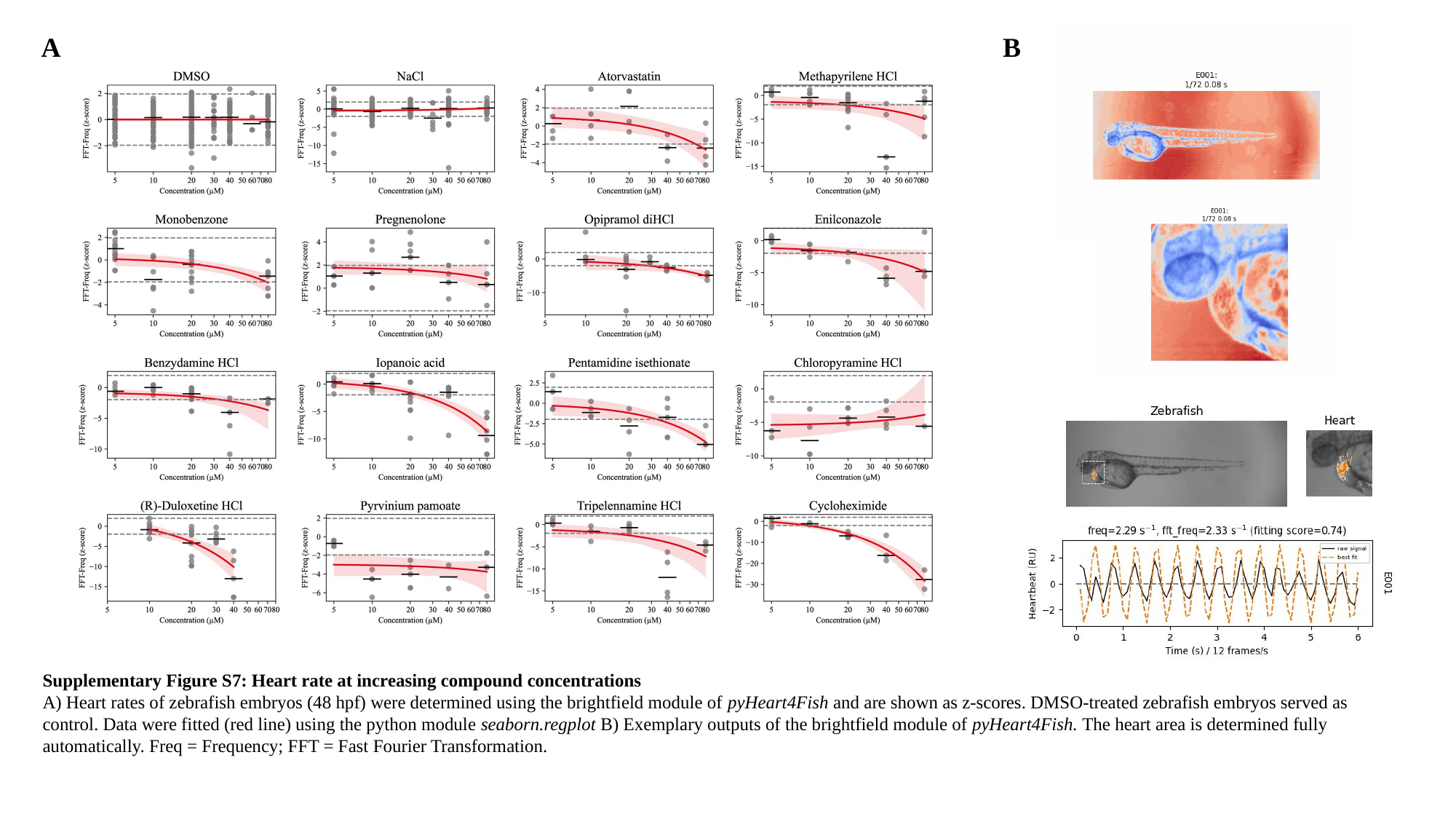

A
B
Supplementary Figure S7: Heart rate at increasing compound concentrations
A) Heart rates of zebrafish embryos (48 hpf) were determined using the brightfield module of pyHeart4Fish and are shown as z-scores. DMSO-treated zebrafish embryos served as control. Data were fitted (red line) using the python module seaborn.regplot B) Exemplary outputs of the brightfield module of pyHeart4Fish. The heart area is determined fully automatically. Freq = Frequency; FFT = Fast Fourier Transformation.
